# Efficacy of targeted drugs in germ cell cancer cell lines with differential cisplatin sensitivity

**DOI:** 10.1371/journal.pone.0178930

**Published:** 2017-06-07

**Authors:** Judith Schaffrath, Hans-Joachim Schmoll, Wieland Voigt, Lutz P. Müller, Carsten Müller-Tidow, Thomas Mueller

**Affiliations:** 1 Department of Internal Medicine IV, Oncology/Hematology, Martin Luther University Halle-Wittenberg, Halle (Saale), Germany; 2 Workgroup Clinical Studies in Oncology, Martin Luther University Halle-Wittenberg, Halle (Saale), Germany; 3 Medical Innovations and Management, Innovation in Oncology, Steinbeis University, Berlin, Germany; 4 Department of Internal Medicine V, Hematology, Oncology and Rheumatology, Heidelberg University Hospital, Heidelberg, Germany; University of South Alabama Mitchell Cancer Institute, UNITED STATES

## Abstract

Testicular germ cell tumors (TGCTs) are the most common malignancies in men between the age of 15 and 35. Although cisplatin-based chemotherapy is highly effective in advanced disease, approximately 20% of patients have an unfavorable prognosis due to primary or acquired cisplatin resistance. For these patients, new therapeutic options are urgently needed. In numerous tumor entities, combinations of monoclonal antibodies or kinase inhibitors with chemotherapy exerted promising preclinical or clinical results, which have led to new treatment concepts. This prompted us to investigate the activity of different targeted agents alone or in combination with cisplatin in a panel of TGCT cell lines.

**Methods:**

The *in vitro* activity of kinase inhibitors targeting mTOR (RAD001), EGFR, HER2/neu, VEGFR (AEE788) and IGF-1R (AEW541) alone or in combination with cisplatin was tested in the cisplatin sensitive TGCT cell lines H12.1 and GCT72 as well as in the resistant cell lines H12.1RA, H12.1D, 1411HP and 1777NRpmet using the sulforhodamin-B-(SRB)-cytotoxicity-assay. To evaluate the activity of the kinase inhibitors, western blot analysis of the targeted receptors and their phosphorylated state was performed before and after exposure to each substance.

**Results:**

The different kinase inhibitors demonstrated significant cell growth inhibition in both cisplatin sensitive and resistant cell lines. The examined cell lines showed different protein expression levels of the targeted receptors. However there was no correlation between the targeted receptor expression and phosphorylation level and the antiproliferative effect of the respective agent. Furthermore, the combination of cisplatin and the kinase inhibitors exerted both additive and antagonistic effects in the studied cell lines.

**Conclusion:**

Our data suggest potential activity of the investigated kinase inhibitors in both cisplatin sensitive and resistant TGCT cell lines as a single agent. However, when combined with cisplatin they did not demonstrate any promising ability to overcome cisplatin resistance in TGCTs.

## Introduction

Testicular germ cell tumors (TGCTs) are the most common malignant solid tumors in young adult men between the age of 15 and 35 with a rising incidence within the last 40 years [1,2]. TGCTs are highly sensitive to chemotherapeutic agents, especially cisplatin, leading to excellent response rates to cisplatin-based chemotherapy even in advanced stages and the rise of the five-year survival rate from 63% to 96% today [3–5]. Therefore, a combination of cisplatin, etoposide and bleomycin (PEB) remains the standard regimen for first line treatment of TGCTs [5]. Despite these favorable response rates, approximately 20–30% of patients cannot be cured by standard therapy and especially those developing cisplatin resistance still have an unfavorable prognosis [6]. The molecular mechanisms behind both the exceptional sensitivity towards cisplatin as well as the development of cisplatin resistance remain unknown.

As a heterogeneous group of tumors, GCTs account for 90–95% of all testicular malignancies and are classified into seminomatous and nonseminomatous germ cell tumors based on their histology [7–9]. Nonseminomatous TGCTs mostly contain both undifferentiated and differentiated elements. The undifferentiated embryonal carcinoma (EC) cells show pluripotential stem cell character and have the potential of differentiating into either extra-embryonal tissues like choriocarcinoma (CC) and yolk sac tumor (YST) or somatic derivatives like teratoma (TE) [7,10,11]. During the course of differentiation, EC cells lose expression of OCT4. OCT4 is an embryonal transcription factor that is uniquely expressed in TGCTs, but whose expression is limited to the germ cell neoplasia in situ (GCNIS) and undifferentiated EC cells, while extra-embryonally and somatically differentiated tissues, i.e. YST, CC and TE lack expression [12,13]. Some data suggest that cisplatin sensitivity depends on the expression of OCT4 and therefore cisplatin resistance is caused by a loss of OCT4 expression [14]. Other approaches of explanation include an increased DNA repair capacity due to alterations in the nucleotide excision repair (NER) system [15], an impaired mismatch repair (MMR) system with increased rates of microsatellite instability in resistant tumor samples [16–18], mutations in the BRAF V600E oncogen (26% in resistant TGCTs versus 1% in sensitive TGCTs) [17] and different mechanisms influencing apoptosis induction such as decreased activation of caspase 9 [19] or significantly higher expression of CCND1 (cyclin D1) in TGCT cell lines with artificially induced cisplatin resistance [20]. Without a full understanding of the mechanisms underlying cisplatin resistance in TGCTs, new therapeutic approaches with the goal of overcoming this resistance have mostly led to disappointing results [21].

During the last decade, the development of new therapeutic agents in the field of oncology focused on the wide spectrum of targeted drugs, both as monoclonal antibodies and so-called “small molecules”, directly inferring into the regulatory mechanisms of the cell by influencing extra- and intracellular signaling cascades. Due to this different mode of action and therefore different side effect profile compared to conventional cytotoxic agents, they are considered to be promising combination partners for the well-known chemotherapeutic substances. In various tumor entities, both the overexpression of receptors and tyrosine kinases such as EGFR, HER2/neu and IGF-1R, the inhibition of tumor cell growth by kinase inhibitors and synergistic effects of combining these inhibitors with conventional chemotherapy were shown [22–25].

RAD001 is an inhibitor of the serin/threoninkinase mTOR (mammalian target of rapamycin) which is integrated in many intracellular signaling pathways. Although no mutations of mTOR itself are known, alterations of mTOR dependent processes have been described in various tumor entities [26,27]. Agents inhibiting mTOR are already well integrated into treatment concepts, e.g. of advanced renal cell carcinoma [28].

AEW541 is a selective, orally applicable inhibitor of IGF-1R (insulin-like growth factor-1 receptor), showing a 27fold higher affinity to this receptor compared to the structurally similar insulin receptor [29]. Although it is not approved for clinical use, it has shown efficacy in Ewing’s sarcoma and multiple myeloma both in vitro and in mouse models and showed additive effects when combined with chemotherapeutic agents in these tumors [30,31]. To our knowledge, no data concerning IGF-1R and mTOR expression in TGCTs is currently available.

AEE788 is a tyrosine kinase inhibitor with multiple target proteins, inhibiting EGFR (epidermal growth factor receptor), HER2/neu (human epidermal growth factor receptor 2) and VEGFR (vascular endothelial growth factor receptor) [32]. Overexpression of EGFR, HER2/neu and VEGFR has been found in many tumor entities, e.g. colon, lung and ovarian cancer, head and neck tumors as well as breast cancer [33,34]. Studies concerning the expression of these receptors in TGCTs have shown conflicting results, with some suggesting an overexpression of EGFR [35,36], HER2/neu [37–39] and VEGFR [40,41] in TGCTs.

The promising preclinical or clinical results of combination therapy in numerous tumor entities prompted us to investigate the activity of different targeted agents in a panel of TGCT cell lines with different cisplatin sensitivity. The kinase inhibitors RAD001, AEW541 and AEE788 were chosen due to their broad spectrum of targeted kinases—also possibly inhibiting escape pathways, especially when combined with each other—and the significance of the inhibited kinases in interfering with apoptosis enhancing mechanisms essential to the efficacy of cisplatin. The aim was to prove a possible ability of these agents to overcome cisplatin resistance.

## Materials and methods

### Cell lines

The following nonseminomatous TGCT cell lines were used: H12.1 [42], H12.1D [14], 1411HP [43], GCT72 [44] and 1777NRpmet [45]. Cell line H12.1RA was derived and isolated from the H12.1 through treatment with ATRA (all-trans retinoic acid) and simultaneous cultivation in conditioned medium of the GCT72 cell line.

The cell lines were maintained as monolayer cultures in RPMI 1640 (Sigma-Adrich, Taufkirchen, Germany) supplemented with 10% fetal bovine serum (Biochrom GmbH, Berlin, Germany) and 1% streptomycin/penicillin (Sigma) and cultures were grown in a 37°C humidified atmosphere containing 5% CO_2_.

### Cytotoxicity assay and drug interaction analysis

Cytotoxicity of cisplatin (Sigma), RAD001, AEW541 and AEE788 (Novartis Pharma GmbH) was determined using the sulforhodamin-B-(SRB)-cytotoxicity-assay. In growth kinetic curves previously defined cell line specific cell numbers were plated in 96-well plates overnight. Subsequently they were treated either with a vehicle control (RPMI 1640) or increasing concentrations of cisplatin (0,01–100 μM) or one of the above enumerated agents (0,001–10 μM) diluted in RPMI 1640 for 96 hours respectively. The SRB assay was carried out as described previously [46]. Trichloroacetic acid was added for fixation, followed by 0.4% SRB. Plates were then washed and the SRB was dissolved by 10 mM Tris buffer and read at 570 nm on a microplate reader. Experiments were performed in three independent series and the mean half maximal inhibitory concentration (IC50) was used to compare cytotoxicity.

To evaluate the effect of combining cisplatin with the kinase inhibitors, cells were seeded into 96-well plates with two plates necessary for every cell line and combination (plate 1: combination of cisplatin and kinase inhibitor; plate 2: cisplatin alone). Leaving the first row as a vehicle control in every plate, the kinase inhibitor with a concentration corresponding to its IC30 value was added to plate 1 from the second row on. Simultaneously, cisplatin in a dilution series was added starting at row 3, leaving row 2 on plate 1 with only the kinase inhibitor as a control sample to determine growth inhibition caused by the kinase inhibitor alone. Cells in plate 2 were only treated with a dilution series of cisplatin (0,01–100 μM) as described above. After 96 hours of incubation, the SRB assay was carried out. To evaluate the effect of combining two agents, a hypothetical curve calculated by normalizing the dose response data points of plate 1 to the inhibitor control (row 2, plate 1) was compared to the dose-response curve for cisplatin alone (plate 2). Synergy can be assumed if the hypothetical curve runs below the dose-response curve for cisplatin and antagonism is indicated if the hypothetical curve runs above the cisplatin curve. In case of additivity both curves are superimposed. The effect of the combination treatment was assessed using the quotient Q of the IC50 values (IC50_control_: IC50_combination_) in both curves. Q = 1 corresponds to an additive, Q < 1 to an antagonistic and Q > 1 to a synergistic effect.

When combining RAD001 with AEW541 or AEE788, RAD001 with a concentration corresponding to its IC30 value was added to a dilution series of AEW541 or AEE788. The following steps and the assessment of the effect of the combination treatment were performed as described above.

### Western blot analyses

Analysis of protein expression was performed using western blot analysis. After protein extraction and measuring of protein concentrations [47], SDS—polyacrylamide gel electrophoresis (SDS—PAGE) and western blotting was performed according to the standard protocol described previously [19] using the antibodies listed in Table 1. Prior to the expression analyses, cells were passaged and suspended in culture medium. After 24 h culture medium was exchanged and after another 24 h total protein was isolated. To investigate the influence of the kinase inhibitors on receptor expression and phosphorylation, cell lines were suspended in culture medium for 24 h and then treated with either one of the kinase inhibitors at a concentration equaling IC30 over 24 hours or culture medium for the same time period.

**Table 1:** Primary antibodies used in the western blot analyses.

Antibody	Commercial Supplier	Monoclonal / Polyclonal	Host Species	Dilution
EGFR	BD Transduction Laboratories	Monoclonal	Mouse	1:500
Phospho-EGFR	BD Transduction Laboratories	Monoclonal	Mouse	1:500
HER2/neu	Cell Signaling Technology	Polyclonal	Rabbit	1:1000
Phospho-HER2/neu (Tyr1221/1222)	Cell Signaling Technology	Polyclonal	Rabbit	1:1000
IGF-1R	Cell Signaling Technology	Polyclonal	Rabbit	1:1000
Phospho-IGF-1R (Tyr1131)	Cell Signaling Technology	Polyclonal	Rabbit	1:1000
mTOR	Cell Signaling Technology	Monoclonal	Rabbit	1:1000
Phospho-mTOR (Ser2448)	Cell Signaling Technology	Polyclonal	Rabbit	1:1000
Phospho-mTOR (Ser2481)	Cell Signaling Technology	Polyclonal	Rabbit	1:1000
VEGFR-2	Cell Signaling Technology	Monoclonal	Rabbit	1:1000
Phospho-VEGFR-2 (Tyr951)	Cell Signaling Technology	Monoclonal	Mouse	1:1000

### Statistical analysis

All experiments were repeated a minimum of three times. Statistical analysis was carried out using Microsoft Excel^®^. When comparing the results of the SRB assays, student's t-test was used to determine significant differences. Statistical significance was assumed at a p-value of < 0.05.

## Results

### Efficacy of cisplatin, RAD001, AEW541 and AEE788 in the TGCT cell lines

**Fig 1:**
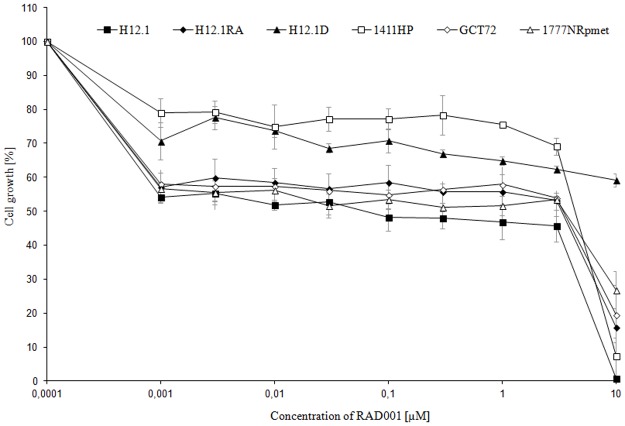
Dose-response curves of RAD001 in TGCT cell lines. (96 h of exposure, n = 3, mean ± SD).

With IC50 values of cisplatin ranging from 0,5 to 10,45 μM, the investigated GCT cell lines exhibited differential cisplatin sensitivity (Table 2). Cell lines H12.1RA, H12.1D, 1411HP and 1777NRpmet showed a relative resistance compared to the cisplatin sensitive cell lines H12.1 and GCT72. The kinase inhibitors RAD001, AEE788 and AEW541 showed activity in all TGCT cell lines and inhibited proliferation in both cisplatin sensitive and resistant cell lines. TGCT cell lines showed a differential sensitivity towards AEW541 with IC50 values ranging from 0,21 μM (1777NRpmet) to 1,80 μM (1411HP). Most cell lines were resistant to AEE788 treatment except H12.1RA (IC50 0,13 μM) which demonstrated pronounced sensitivity. Interestingly, the dose-response curves of RAD001 showed an untypical pattern (Fig 1). They were characterized by a plateau starting at a concentration of 1 nM and spanning a concentration range which included the IC50 values of most cell lines. Therefore, the IC50 did not qualify as a suitable parameter for comparing the sensitivity of the cell lines and the mean IC50 values show large standard deviations. A comparison of RAD001 activity at the specific concentration of 1 nM revealed a growth inhibition of about 45% in cell lines H12.1, H12.1RA, 1777NRpmet and GCT72. The cell lines 1411HP and H12.1D were less sensitive towards RAD001 leading to growth inhibition of 20 and 30%, respectively.

**Table 2:** IC50 values (μM) of cisplatin, RAD001, AEW541 and AEE788 after 96 h of exposure (n = 3, mean ± SD).

	CDDP	RAD001	AEW541	AEE788
**H12.1**	0,5 (±0,11)	0,17 (±0,23)	0,50 (±0,05)	5,11 (±0,49)
**H12.1RA**	8,68 (±1,76)	3,43 (±0,42)	0,24 (±0,02)	0,13 (±0,02)
**H12.1D**	10,45 (±3,28)	>10	0,83 (±0,33)	> 10
**1411HP**	4,70 (±0,44)	4,37 (±0,29)	1,80 (±0,19)	4,54 (±0,24)
**GCT72**	0,58 (±0,04)	3,34 (±0,74)	0,85 (±0,82)	4,96 (±0,18)
**1777NRpmet**	1,64 (±0,74)	3,54 (±0,27)	0,21 (±0,10)	5,99 (±0,18)

### Efficacy of a combination of cisplatin and kinase inhibitors RAD001, AEW541 and AEE788

**Fig 2:**
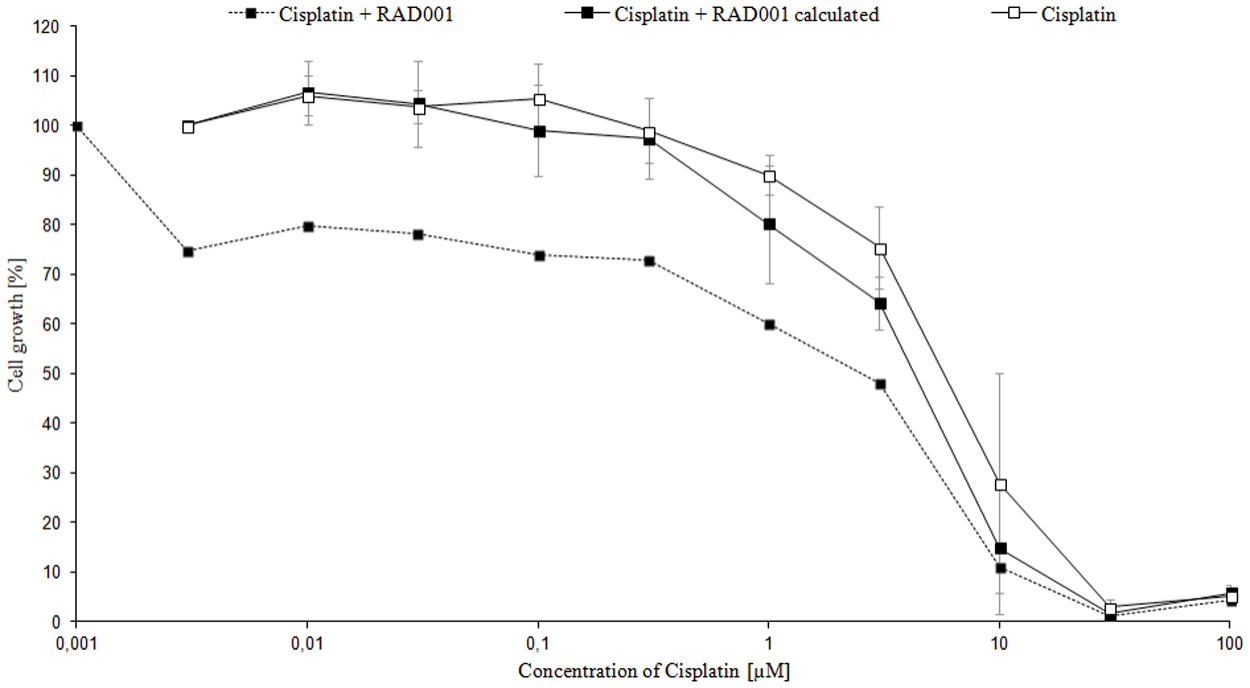
Dose-response curve of cisplatin compared to the combination of cisplatin and RAD001 in the cell line 1411HP. (96 h of exposure, n = 3, mean ± SD).

For each cell line combination assays with cisplatin and each of the examined kinase inhibitors were performed. A hypothetical curve was calculated from dose-response data points of each combination treatment enabling analyses of the impact of kinase inhibitors on cisplatin antitumor activity (Fig 2). The combination of cisplatin and kinase inhibitors showed both additive and antagonistic effects in the studied cell lines, yet no significant synergistic effect was observed. In the cisplatin resistant cell line 1411HP there was dominantly an additive effect, however with a trend to synergy for the combination of cisplatin and RAD001. In the H12.1D significant antagonistic effects of cisplatin and both AEW541 and AEE788 were observed. Results of the combination assays are summarized in Table 3.

**Table 3:** Combination assays of cisplatin and kinase inhibitors RAD001, AEW541 and AEE788.

	Q_CDDP + RAD001_	Q_CDDP + AEW541_	Q_CDDP + AEE788_
**H12.1**	0,67	1,21	0,86
**H12.1RA**	1,06	0,91	0,81
**H12.1D**	1,02	0,75 (*)	0,51 (*)
**1411HP**	1,47	0,69	0,96
**GCT72**	1,39	1,92	1,23
**1777NRpmet**	0,64	1,29	0,53

Stated are the quotient Q of the IC50 values (IC50_control_: IC50_combination_). Q = 1 corresponds to an additive, Q < 1 to an antagonistic and Q > 1 to a synergistic effect.

^(^*^)^ indicates a significant effect. (n = 3, mean ± SD, student’s t-test)

### Efficacy of a combination of RAD001 with AEW541 and AEE788

Based on the interacting pathways downstream of the kinases inhibited by RAD001, AEW541 and AEE788 we also investigated the effects of a dual, vertical blockade using two kinase inhibitors in the cisplatin sensitive cell line H12.1 and the cisplatin resistant cell line 1411HP.

As shown in Table 4, we observed additive effects in both cell lines. When combining RAD001 and AEE788 a synergistic tendency was seen at lower concentrations (IC20).

**Table 4:** Combination assays of RAD001 and AEW541 / AEE788.

		Q_AEW541 + RAD001_	Q_AEE788 + RAD001_
**H12.1**	IC20		2,08
	IC50	1,11	1,18
**1411HP**	IC20		2,03
	IC50	1,33	1,09

Stated are the quotient Q of the IC50 values (IC50_control_: IC50_combination_). Q = 1 corresponds to an additive, Q < 1 to an antagonistic and Q > 1 to a synergistic effect. (n = 3, mean ± SD, student’s t-test)

### Expression and phosphorylation of targeted receptors

**Fig 3:**
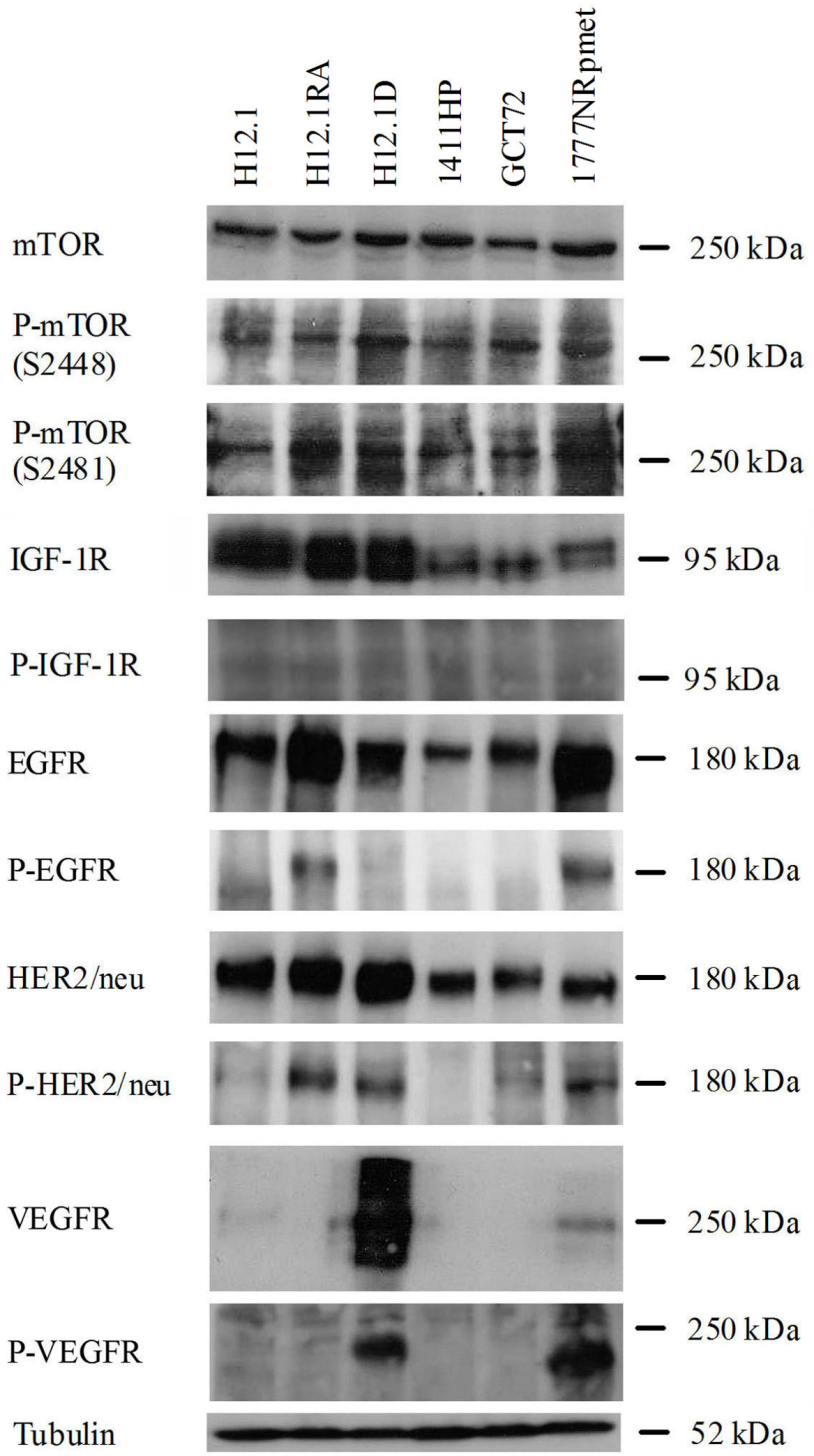
Expression and phosphorylation of EGFR, HER2/neu, VEGFR, IGF-1R and mTOR in all cell lines.

Both the expression and phosphorylation of the receptors targeted by RAD001 (mTOR), AEW541 (IGF-1R) and AEE788 (EGFR, HER2/neu und VEGFR) was examined in all cell lines using western blotting (Fig 3). The TGCT cell lines showed different expression and phosphorylation profiles of the targeted receptors. All investigated cell lines expressed EGFR with an increased expression in the cell lines H12.1RA and 1777NRpmet, which also showed phosphorylation of the receptor. HER2/neu was also detected in all cell lines with increased expression in H12.1, H12.1RA and H12.1D. Phosphorylation of HER2/neu was found in H12.1RA, H12.1D and 1777NRpmet. VEGFR was strongly expressed in the H12.1D as well as in the 1777NRpmet with phosphorylation also limited to those two cell lines. Given the expression and specific phosphorylation pattern of receptors, sensitivity to AEE788 could be expected in H12.1, H12.1RA, H12.1D and 1777NRpmet. However, a correlation between the receptor expression and phosphorylation and the effectiveness of AEE788 was only seen in H12.1RA.

IGF-1R was found in all cell lines with the strongest expression in H12.1, H12.1RA and H12.1D, therefore showing no clear correlation between receptor expression and sensitivity to AEW541. The comparison of the expression was complicated by the formation of multiple bands. The phosphorylated form of IGF-1R could not be detected sufficiently in our western blot analyses. This was in contrast to the good response of all cell lines to treatment with AEW541.

Both mTOR and its activated form, investigated using two antibodies against both phosphorylation sites S2448 und S2481, were detected consistently in all examined cell lines. This corresponds at least in part to the observed activity of RAD001 in all cell lines at a very low concentration.

### Receptor expression and phosphorylation after treatment with kinase inhibitors

**Fig 4:**
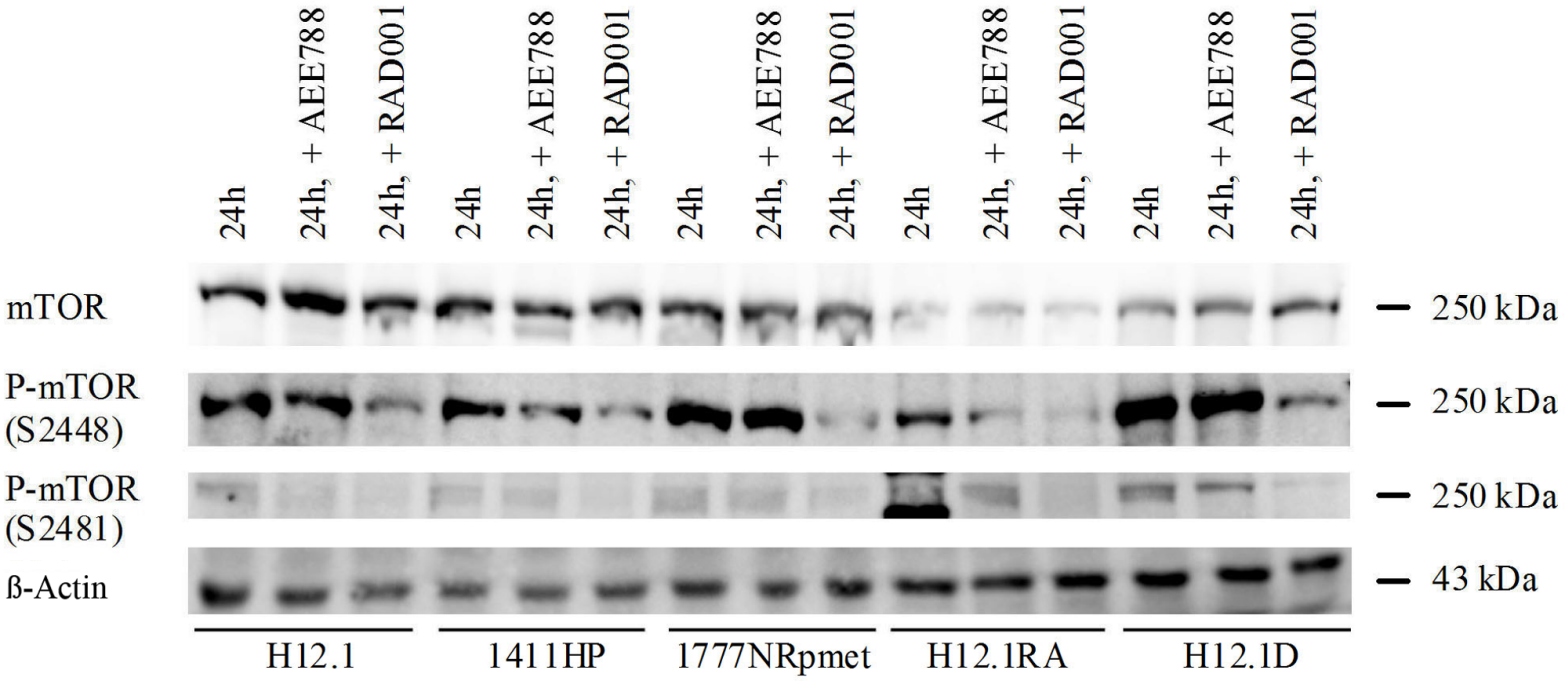
Expression and phosphorylation of mTOR after treatment with AEE788 (IC30) and RAD001 (30 nM) over 24 h compared to untreated controls. The additional band at P-mTOR S2481 (H12.1RA, 24 h) is caused by the marker.

Due to the growth inhibition caused by RAD001 in all cell lines and the central role of mTOR in intracellular signaling pathways regulating cell growth downstream of IGF-1R, EGFR, HER2/neu and VEGFR, we then concentrated on the effects of the tyrosine inhibitors in mTOR expression and phosphorylation. Treatment with RAD001 did not influence the expression of mTOR but led to an inhibition of phosphorylation on both phosphorylation sites S2481 und S2448 in all investigated cell lines. Interestingly, treatment with AEE788 also led to a decreased activation of mTOR in some cell lines (Fig 4).

## Discussion

Cisplatin resistance remains a major problem in the treatment of patients with nonseminomatous germ cell tumors. At this point, neither the mechanisms underlying this resistance nor promising therapeutic options have been identified. The introduction of targeted drugs such as monoclonal antibodies and kinase inhibitors has led to encouraging results for many other tumor entities. Due to their differing mechanism of action and side effect profile, a combination with traditional chemotherapeutic agents is both feasible and reasonable, showing additive and synergistic effects in both preclinical and clinical studies [24,25]. Therefore, we aimed to investigate the expression and activation of the drugable targets EGFR, HER2/neu, VEGFR, IGF-1R and mTOR, the efficacy of kinase inhibitors targeting these proteins and their influence on cisplatin sensitivity in both cisplatin sensitive and resistant TGCT cell lines.

The first goal was to characterize the TGCT cell lines according to their sensitivity towards cisplatin and the examined kinase inhibitors. The IC values acquired for cisplatin were consistent with previously published data [14]. H12.1RA, which was obtained after treatment of the H12.1 with ATRA, leading to a somatic, neural differentiation with loss of the characteristic surface antigen SSEA-3 and SSEA-4 (stage-specific embryonic antigen-3 and 4), was also cisplatin resistant [48,49].

Cytotoxicity assays for RAD001 showed a similar sensitivity of all cell lines with dose-response curves showing a plateau spanning a concentration range which included the IC50 starting around a concentration of 0,001 μM. This characteristic curve progression has been described for many other tumor entities [50]. Compared to IC50 values of *in vitro* tests in other malignancies, the investigated TGCT cell lines showed a relative resistance towards RAD001. While the most sensitive TGCT cell line H12.1 exhibited an IC50 of 170 nM and the H12.1D did not reach IC50, in a trial on 48 different tumor cell lines 71% of the treated cell lines had an IC50 < 0,1 μM and in sensitive renal cell cancer cell lines an IC50 of 0,78±0,23 nM has been described [50,51]. Yet, as already mentioned above, due to the plateau in the dose response curves the IC50 value does not serve as a suitable parameter for comparison. Since this plateau is reached at about 0,001 μM in the cell lines H12.1, H12.1RA, GCT72 and 1777NRpmet, these TGCT cell lines should be considered RAD001 sensitive.

The overall small differences in sensitivity towards RAD001 in the investigated cell lines are reflected in the equal expression and activation of mTOR. Furthermore, there was neither a difference in sensitivity nor in expression and activation of mTOR between cisplatin sensitive and resistant cell lines. While the inhibition of mTOR expression and phosphorylation at S2448 by RAD001 has been proven in multiple cancer cell lines [52–54], an increase in phosphorylation at S2481 in osteosarcoma has also been described [55]. In the examined TGCT cell lines the expression of mTOR remained uninfluenced by treatment with RAD001, yet the inhibition of phosphorylation on both sites S2448 and S2481 proves the specific mechanism of action of RAD001 in TGCTs.

Another goal of our study was to examine the influence of kinase inhibitors on cisplatin sensitivity, especially in cisplatin resistant TGCT cell lines. The synergistic effect of combining RAD001 and cisplatin has been shown in many different tumor entities, both *in vitro* [56,57] and in mouse models with human xenografts [50]. Furthermore, overcoming of cisplatin resistance by the addition of RAD001 has been described in cisplatin resistant gastric cancer cells by Ying *et al*. [58]. In our cell lines, a divergent response with mostly additive effects was observed. However, no clear ability to overcome cisplatin resistance could be demonstrated. Nevertheless, in the resistant 1411HP there was an additive effect with a trend to synergy for the combination of cisplatin and RAD001. In addition, RAD001 turned out to be the best combination partner among the targeted drugs investigated in this study. Therefore, the inhibition of the PI3K/AKT/mTOR-*pathway* might be a promising target to overcome cisplatin resistance. In their studies of lung cancer cells, Beuvink *et al*. could show that RAD001 inhibits the cisplatin induced induction of p21 by p53. In case of a p53-wildtype both the p21-induced cell cycle arrest and the inhibition of pro-apoptotic caspases (procaspase 3, caspase 8) by p21 are averted [59]. Due to the low mutation rate of p53 in TGCTs, this mechanism might be transferable to TGCTs and offers possibilities for further studies [60].

Although at this point no IGF-1R inhibiting substance has been approved for clinical use due to severe side effects, we also investigated the IGF-1R inhibitor AEW541 since its cytotoxic activity has been proven in several *in vitro* analyses, e.g. in Ewing’s sarcoma, multiple myeloma and gastrointestinal stromal tumors and we could not find any data concerning IGR-1R expression in TGCTs [30,61,62]. In our cytotoxicity assays, AEW541 alone inhibited cell growth in all cell lines with IC50 values between 0,21 μM (1777NRpmet) and 1,8 μM (1411HP). In pancreas carcinoma cells (IC50 0,34–2,73 μM) and ovarian cancer cells (IC50 5–15 μM) similar results have been reported [63,64].

The lack of detection of the phosphorylated IGF-1R in our western blot analyses despite expression of the receptor and efficacy of AEW541 may have had methodological reasons. In their studies on gastrointestinal stromal tumors and pancreatic cancer cell lines, both Tarn *et al*. and Moser *et al*. could only detect both the phosphorylated form of IGF-1R and its inhibition by AEW541 after stimulation with IGF-1 [61,65]. Besides that, our cell lines were maintained in RPMI 1640 supplemented with 10% fetal bovine serum. In their studies on AML cell lines, Tazzari *et al*. could show that while expression of IGF-1R remains uninfluenced by AEW541, phosphorylation of the receptor is inhibited. They could further show that a change in serum concentration (10% vs. 2%) led to an increased activity of AEW541 in the lower concentrated serum. The authors attributed this finding to the growth factors contained in fetal bovine serum, decreasing the efficacy of AEW541 by stimulating antiapoptotic signaling pathways [66].

Concerning the correlation between expression and phosphorylation of IGF-1R and the efficacy of AEW541 contradictory data have been published. While Browne *et al*. described a relative resistance towards AEW541 in breast cancer cell lines with increased expression and phosphorylation of IGF-1R [67], this correlation was not found in other studies [68]. Yet Mukohara *et al*. could demonstrate that in their investigated breast cancer cell lines the expression of IRS-1 (insulin receptor substrate 1) correlates with the response to AEW541, identifying IRS-1 expression of a marker for AEW541 efficacy independent of IGF-1R expression [68]. This might also explain the homogeneous response towards AEW541 in the TGCT cell lines despite differential IGF-1R expression. In addition, this mismatch might also be explained by IGF-1R independent effects, especially an inhibition of the insulin receptor (IR) due to the structural similarity of IGF-1R and IR. Although a high selectivity of AEW541 towards IGR-1R has been shown (IC50_IGF-1R_ = 0,09 μM, IC50_IR_ = 2,30 μM), part of the cytotoxic potential may also be caused by inhibition of the IR [29,69].

In *in vitro* studies, different IGF-1R inhibiting substances have led to additive and synergistic effects when combined with cisplatin, e.g. the IGF-1R antibodies MK-0646 in endometrial carcinoma and R1507 in small cell lung cancer [70,71]. The combination of cisplatin and AEW541 has led to antagonistic effects in Ewing’s sarcoma and synergistic effects in ovarian cancer cells [30,63]. In our analyses, AEW541 showed a different effect on cisplatin sensitivity in the investigated cell lines and was not able to overcome cisplatin resistance. Due to the sparse data on the role of IGF-1R in TGCTs, the reason for these divergent results remains unclear.

The efficacy of an inhibition of EGFR and HER2/neu in TGCTs has been demonstrated in *in vivo* studies in mice using Lapatinib [72]. The dual tyrosine kinase inhibitor AEE788 led to a growth inhibition in all investigated TGCT cell lines, yet with the exception of the cell line H12.1RA they all showed a relative resistance (IC50 between 4,54 μM and > 10 μM). Park *et al*. found IC50-values between 0,12 μM and 0,96 μM in squamous cell carcinoma [73].

There is conflicting data concerning the expression and activity of EGFR, HER2/neu and VEGFR in TGCTs with no clear proof of an overexpression of any of the investigated receptors [35,39]. The fact that the H12.1RA shows somatic differentiation conforms with the results of Mandoky *et al*., showing that HER2/neu expression was limited to the teratoma and choriocarcinoma components of mixed TGCTs [38]. In contrast, Juliachs *et al*. could detect expression of EGFR and HER2/neu in all components of TGCTs independent of their differentiation [72].

Because numerous studies have shown increased cisplatin sensitivity after inhibition of EGFR, HER2/neu or VEGFR in different tumor entities, we performed combination assays despite the relative resistance of our investigated cell lines towards AEE788 [74,75]. Even with low sensitivity towards AEE788 in salivary adenoid cystic carcinoma, cisplatin efficacy was enhanced by AEE788 [76]. In most TGCT cell lines we observed an antagonistic tendency; only in the 1411HP and GCT72 we saw an additive effect. Similar findings were published by Perry *et al*., showing that an inhibition of EGFR and HER2/neu by Lapatinib did not influence the efficacy of cisplatin in TGCTs, regardless of cisplatin sensitivity [77]. An explanation for the antagonistic effects of AEE788 and cisplatin might be offered by the studies of Yamaguchi *et al*. on non small cell lung cancer. They could prove that an inhibition of the EGFR pathway by Gefitinib also inhibits caspase independent cell death, which can be induced by cisplatin. Since they also detected an activation of EGFR and its downstream targets AKT and ERK by cisplatin, they postulated that cisplatin might induce caspase independent cell death via EGFR associated pathways—a mechanism that is repressed by EGFR inhibition [78]. Finally, the application sequence of cisplatin and kinase inhibitors can influence the result of combination treatment, possibly also leading to antagonistic effects. In studies with gemcitabine, cisplatin and lapatinib in bladder cancer cell lines, the optimal sequence consisted of lapatinib before and during gemcitabine and cisplatin, perhaps due to a cell cycle arrest caused by lapatinib [79].

Finally, we also tested the effects of a combination of two kinase inhibitors, thereby causing a vertical blockade of the involved signaling pathways with mTOR being an important downstream target of the inhibited growth factor receptors IGF-1R, EGFR, HER2/neu and VEGFR. Furthermore, a combined blockade of multiple kinases at different levels within the signaling pathways eliminates possible compensating mechanisms such as an increased activation of upstream targets. In vitro, a simultaneous application of RAD001 and AEW541 has shown additive and synergistic effects in both multiple myeloma and hepatocellular carcinoma [80,81]. In our study we could show additive effects in TGCT cell lines. This might be explained by a dual blockade of the PI3K/AKT/mTOR-*pathway*, since an inhibition of IGF-1R by AEW541 leads to decreased phosphorylation of AKT [63,82]. An additive effect could also be observed when RAD001 was combined with AEE788 in our cell lines. In renal cell and prostate cancer as well as glioblastoma a combination of RAD001 with AEE788 has also led to additive and synergistic effects [83–85]. Additionally, in our western blot analyses AEE788 also led to a decreased phosphorylation of mTOR and therefore seems to also influence the PI3K/AKT/mTOR-*pathway*. Combining different kinase inhibitors could be a strategy to overcome chemotherapy resistance but it may not only lead to an increased efficacy but also to severe side effects. For example, a simultaneous application of RAD001 and AEE788 in glioblastoma patients was reported to cause significant thrombocytopenia [86].

In sum, our data show that the investigated kinase inhibitors RAD001, AEW541 and AEE788 are effective in TGCT cell lines independent of their cisplatin sensitivity. However, when combined with cisplatin they do not demonstrate promising ability to overcome cisplatin resistance in these cell lines. No correlation between the receptor expression and phosphorylation and the effectiveness of the agents was observed. Therefore, these targeted drugs do not show potential in the treatment of cisplatin refractory nonseminomatous germ cell tumors.
